# Odontogenic tumours: A review of 266 cases

**DOI:** 10.4317/jced.50949

**Published:** 2013-02-01

**Authors:** Ahmed O. Lawal, Akinyele O. Adisa, Adeola A. Olusanya

**Affiliations:** 1 FMCDS. Lecturer/Consultant. Department of Oral Pathology, College of Medicine, University of Ibadan, Nigeria; 2 FMCDS. Lecturer/Consultant. Department of Oral and Maxillo-facial Surgery, College of Medicine, University of Ibadan, Nigeria

## Abstract

Objectives: The aim of this study was to examine the relative frequency of odontogenic tumours at a tertiary hospital in Ibadan, as well as to study the various histologic types based on WHO 2005 classification and to compare results from this study with those of previous studies. Study design: The records of the Oral Pathology Department of University College Hospital were reviewed. Lesions diagnosed as odontogenic tumours were categorized into four groups based on WHO 2005 classification and were analyzed for age, sex and site using SPSS for Window (version 18.0; SPSS Inc. Chicago, IL) and frequency tables were generated. Results: Two hundred and sixty six (41.7%) cases of odontogenic tumours were seen. The mean age of occurrence was 32.6 (±15.815) years (range3-82 years) and peak age was in the third decade of life. Eleven (4.1%) malignant odontogenic tumours were seen. Ameloblastoma with 65.4% of cases was the most common odontogenic tumour followed by fibromyxoma (14.7%), no case of odontoma was seen in this series. Conclusion: The findings were mostly similar to those of African and Asian series and showed variations from reports from the Americas. The reason for the disparity in African and American series needs further investigations.

** Key words:**Odontogenic tumour, classification, Nigeria.

## Introduction

Odontogenic tumours (OTs) constitute a wide range and diverse kind of lesions derived from tooth forming apparatus and its reminants (1). OTs originate from epithelium or ectomesenchyme or from both, showing varying degrees of inductive interaction between these embryonic components of the developing tooth germ (2).

The relative frequency of OTs obtained from studies from different parts of the world, have varied widely. Some authors have reported that OTs are rare with a relative frequency of 1% (3), while others have reported OTs constitute up to 32% (4) of jaw lesions. Furthermore, whilst American (2,3) studies showed odontomas as the most common OT, studies from Africa (4) and Asia (1) have shown ameloblastoma to be, overwhelmingly, the most common OT. These disparities have being suggested to be due to the differences in terminology and classification and also, possibly due to racial and or genetic differences in the occurrence of the various types of OTs (3).

Although, many reports on OTs are available from literature, (1,2,3,4) most of these studies were carried out before WHO classification of 2005 (5) which included Keratocystic odontogenic tumour (KCOT) as an odonto-genic tumour. The aim of this study was to examine the relative frequency of OT seen at the Oral Pathology Department of the University College Hospital Ibadan, as well as study the various histologic types based on WHO 2005 classification and to compare results from this study with those of previous studies.

## Material and Methods

The records of the Oral Pathology Department of University College Hospital Ibadan were reviewed over a 21 year period (1990-2011). Lesions diagnosed as OTs were categorized into four groups based on WHO 2005 classification (5); Group 1 were malignant tumours, Group 2 Odontogenic epithelium with mature, fibrous stroma without odontogenic ectomesenchyme, Group 3 Odontogenic epithelium with odontogenic ectomesenchyme, with or without hard tissue formation and Group 4 Mesenchyme and/or odontogenic ectomesenchyme with or without odontogenic epithelium and were analyzed for age, sex and site using SPSS for Window (version 18.0; SPSS Inc. Chicago, IL) and frequency tables were generated.

## Results

A total of six hundred and thirty eight jaw lesions were diagnosed during the study period, out of these, two hundred and sixty six (41.7%) were OTs. The mean age of occurrence was 32.6 (±15.815) years (range 3-82 years) and peak age was in the third decade of life. Odontogenic tumours occurred more in the mandible with mandible: maxilla ratio of approximately 5:1 and 7 cases were seen in soft tissue. OTs had a slight male preponderance with a male: female of 1.2:1. of the 266 seen, 255 (95.9%) were benign, while 11(4.1%) cases of malignant Odontogenic tumours were seen.

 Table 1 shows demographic distribution of OTs according to the WHO 2005 classification. The Group 2 lesions were the most common odontogenic tumours with 195 (73.3%) lesions seen in this category. Ameloblastoma with a total 174 cases representing 65.4% of all OTs and 89.2% of Group 2 lesions was the most common OT. Ameloblastoma had an obvious predilection for the mandible with 93% of solid ameloblastoma cases occurring in the mandible while 89.7% of cystic ameloblastoma occurred in the mandible. The mean age of the solid ameloblastoma was 34.4 (±15.2) years while that of cystic ameloblastoma was 25.6 (± 8.7) years and both had a peak age of occurrence in the third decade of life.

**Table 1:**
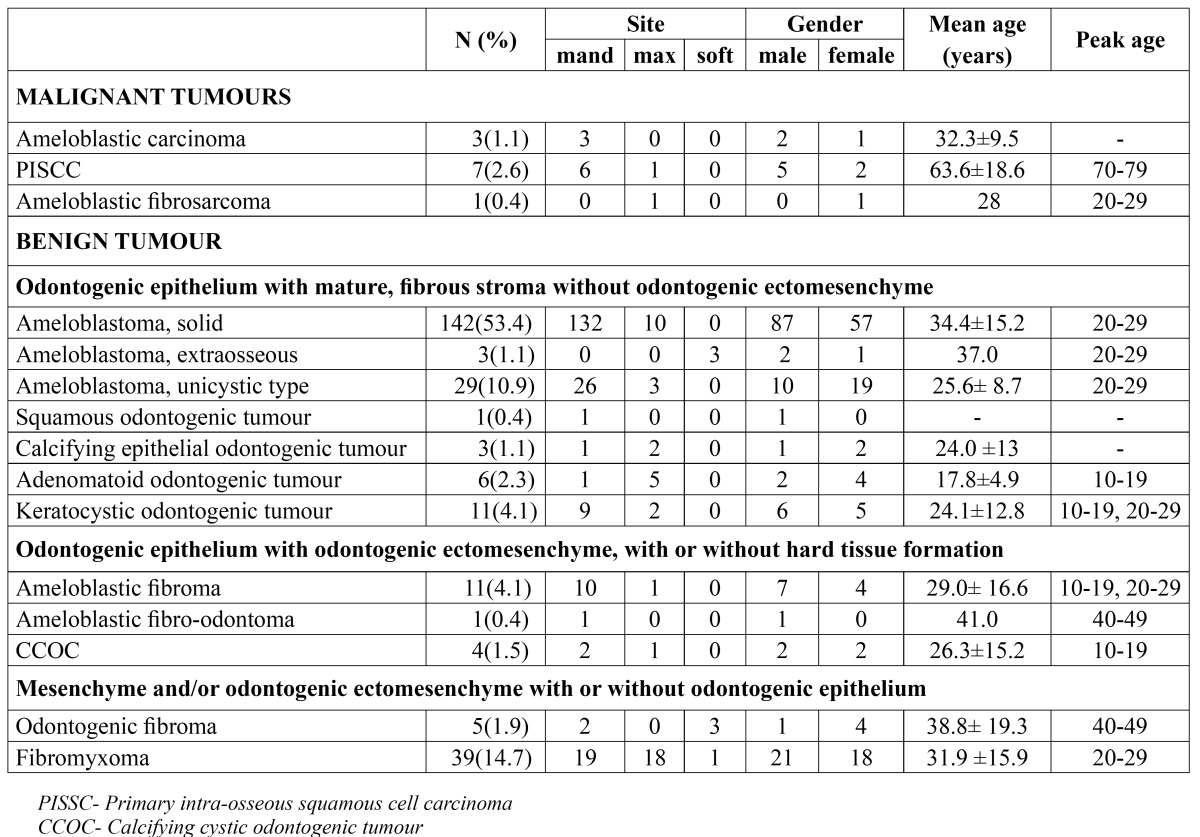
Demographic distribution of odontogenic tumours.

KCOT was the second most common lesion in the Group 2 category of odontogenic tumours and third most common odontogenic tumour. A total of 11 cases of KCOT were seen, which represented 4.1% of odontogenic tumours and 5.6% of the Group 2 lesions. KCOT had a marked predilection for the mandible with mandible: maxilla ratio of 4.5:1 but had a slight male preponderance with a male: female ratio of 1.2:1. KCOT had a bimodal peak age of occurrence in the second and third decades of life with a mean age of 24.1 (±12.8) years. Six cases of adenomatoid odontogenic tumor (AOT) were seen in this series with a predominant maxillary presentation (mandible: maxilla ratio of 1:5) and a female gender predilection (male: female ratio = 1:2). Other lesions categorized in the Group 2 lesions (CEOT, SOT) were seen occasionally and all together accounted for 1.5% of odontogenic tumours and 2.1% of Group 2 lesions.

Group 4 lesions were the second most common category of OTs with 44 (16.6%) cases. Fibromyxoma, which had 39 cases, representing 14.7% of OTs and 88.6% of the Group 4 lesions, was the most common lesion in this Group and second most common OT. Fibromyxoma had no obvious site or gender predilection with a mandible: maxilla ratio of 19:18 and male: female ratio of 7:6.

Group 3 lesions with 16 (6%) of case were the third commonest group of lesions in this series. Ameloblastic fibroma which was the commonest lesion in this group had a mean age of 29.0 (± 16.6) years and had a bimodal peak incidence in the second and third decades of life. Ameloblastic fibroma had an obvious mandibular preponderance with 90.9% of cases occurring in the mandible. A case of ameloblastic fibro-odontoma was seen in mandible of a 41 year old male patient.

Group 1 lesions were the least common group of OTs accounting 4.1% of OTs. Primary intra-alveolar squamous cell carcinoma (PISCC) was the most common malignant OT seen in this series. Seven cases of PISCC which was 2.6% of all OTs and 63.6% of malignant OTs were seen. PISCC had a mandibular preponderance (mandible: maxilla ratio of 6:1) and a male: female ratio of 5:2 with a mean age of 63.6±18.6 years and peak age of occurrence in 70-79 age group. Ameloblastic carcinoma occurred exclusively in the mandible, had a male: female ratio of 2:1 and a mean age of 32.3 (±9.5) years with one case each in the third, fourth and fifth decades. The only case of ameloblastic fibro-sarcoma was seen in the maxilla of a 28 year old woman.

## Discussion

The fact that most studies considering demography of OTs were carried out prior to the reclassification of OTs (1,2,3,4) by WHO in 2005 and the variations in the terminology and classification by previous authours make comparison with other studies quite challenging ( Table 2). Although, many non-African studies have claimed that OTs are a relative rare group of lesions, African studies seem to contradict this notion. Fernandes et al (6) in Brazil, Buchner et al (7) in California and Mosqueda-Taylor et al (2) in Mexico, reported relative frequencies of OTs to be 1.78%, 1.2% and 2.5% respectively. In contrast, Chidzonga et al (8) in Zimbabwe, Ladeinde et al (9) in Lagos and Adebayo et al (4) in Kaduna reported relative frequencies of 8.6%, 9.6% and 32% respectively. The relative frequency of 41% gotten from this study is possibly the highest reported in literature and may be due to the fact that OTs are relatively more prevalent in Africans. Moreover, the relative frequency of OTs in this study was gotten from comparison with tumours and tumour like lesions of the jaws as compared to other studies whose comparison was based on all biopsies. Adebayo et al (4) and Arotiba et al (10) both of whom compared OTs with jaw tumours and tumour like lesions, also got relatively high frequencies of OTs (32% and 30% respectively).

**Table 2:**
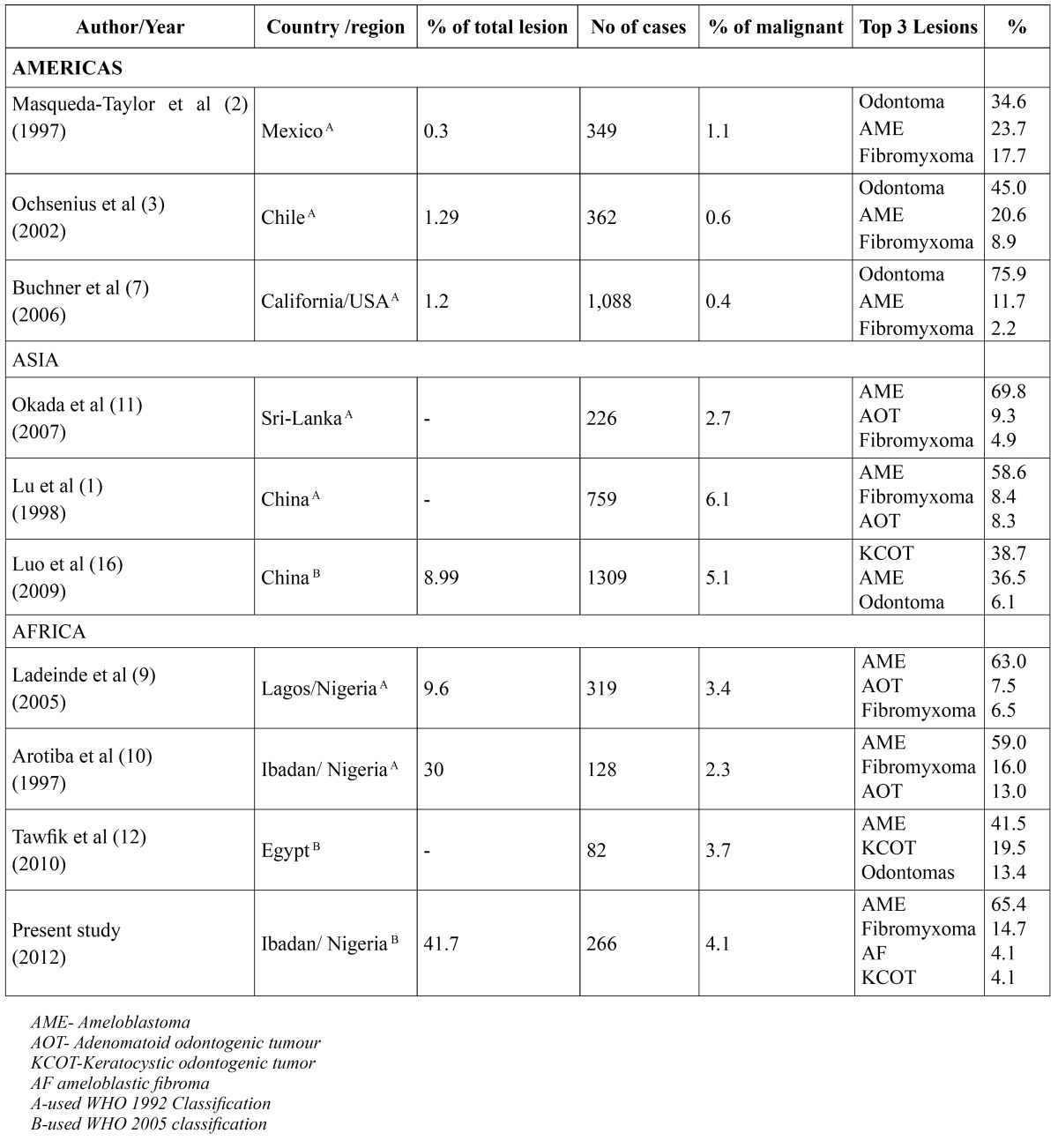
Geographical variations in odontogenic tumours.

Previous studies from Asia and Africa have reported ameloblastoma to be the most common OT (10). Young Lu et al (1) and Okada et al (11) from the Asian continent showed in their series that ameloblastoma represented 58.6% and 68.9% of OTs respectively while Ladeinde et al (9), Adebayo et al (4) Chidzonga et al (8) and Tawfik (12) all from Africa reported that ameloblastoma represented 63%,73%,79.1%, and 41.5% of OTs respectively. In this study, ameloblastoma accounted for 65.4% of OTs which was in conformity with African and Asia studies.

On the contrary, studies from the Americas have shown that odontomas are the commonest OT. Buchner et al (6) in California USA, Mosqueda-Taylor et al (2) in Mexico, Ochsenius et al (3) in Chile all found odontomas to be the most common OT (representing 75.9%, 34.6% and 44.7% respectively), however, Fernandes et al (6) in Brazil found in their study that ameloblastoma with 45.2% of their cases was the most common OT. It should be observed that no case of odontomas was reported in this study which was in contrast with most other studies from Americas (2,6,7), Asia (1,11) and Africa (8,9,12). This may be due to the fact that many odontomas may have been diagnosed and excised by private and secondary dental care providers without subjecting them to histological testing. Furthermore, those diagnosed in our centre, were mainly incidental radiological findings and patients declined appointments for surgical excision and histological testing. However, it should be noted that, Arotiba et al (10) in a previous study from the same centre, also did not find any case of odontomas in their series.

There is yet to be a consensus on the possible reason(s) for the wide disparity in the occurrence of ameloblastomas and odontomas across the continents; some authors have suggested that the Asian and African cases of odontomas may be under reported as orthopanthomogram is yet to be routine in clinical examination in some of these countries and some cases may not be sent for routine histological examination. The suggestion that racial and genetic factors may account for the wide geographical variation in occurrence of OTs remains unproven (3).

Ameloblastoma had an obvious mandibular predilection in this study with 93% of solid ameloblastoma occurring in the mandible while 7% occurred in the maxilla, similarly, 89.7% of cystic ameloblastomas occurred in the mandible. This finding was similar to Young Lu et al (1) in China, Chidzonga et al (8) in Zimbabwe and Arotiba et al (10) in Nigeria who reported 92.8%, 95.7% and 91% of mandibular occurrence for ameloblastoma respectively. The mean age for solid ameloblastoma (34.4 years) was in agreement with previous studies, (3,8) but at variance with some others (4,6,8). Although Reichart et al (13) suggested that ameloblastoma tend to occur at an early age in developing countries when compared with developed ones, this study and some others seems (9,10) to contradict this notion.

The finding that fibromyxoma was the second most common OT was corroborated by previous studies from Kaduna (4) and Ibadan but Ladeinde et al (9) in Lagos found AOT to be the second most common OT. Our finding of a male slight predilection for fibromyxoma was in contrast to most studies (2,3,4,14) which reported a female preponderance, while a few studies found no gender predilection only few previous series have reported a male predilection (4).

AOT with a maxillary predilection and female preponderance was similar to a previous finding by Arotiba et al (15) in Lagos though it was less common (2.3%) in this series than in a series from Lagos. The mean age of 17.4 years was similar to that from Arotiba et al (15) but lower than the 22.6 years gotten from a Chinese series (1). KCOT which was included in the 2005 WHO classification (5) of OT represented 4.1% of OT which was lower than the 19.5% reported by Tawfik (12) in Egypt and the 38.7% reported by Luo (16) in Chinese who found KCOT to be the most common OT is their series. Most other studies were done before KCOT was included as an OT and did not include KCOT in their series. KCOT was seen more in the mandible and had a slight male preponderance which was in conformity with reports from other series (12,16).

Malignant OTs accounted for 4.1% of cases which was in agreement with most African and Asian series but higher than series from the Americas. Ladeinde et al (9) in Lagos Lu et al (1) in China got 3.4% and 6.1% respectively while Fernedez et al (6) in Brazil, Ochsenius et al (3) in Chile and Buchner et al (7) in Califor-nia USA, reported 0.6%, 0.6% and 0.4% respectively.

The results gotten from this study shows that OTs are not rare lesions in Africans and confirms that ameloblastoma was the commonest OT in Africans. The findings were mostly similar to those of African and Asian series and showed variations from reports from the Americas. KCOT reported in this study was relative rarer than those previously reported and malignant OTs were relatively common when compared with American series. The reasons for the disparity in African and American series and the suggestion that racial and genetic factors might account for these differences needs further investigations.
